# The FLY-project: study protocol for mixed methods research to explore the complex social dynamics of sustainable food-related lifestyles in youth in practical education

**DOI:** 10.1186/s40795-023-00757-2

**Published:** 2023-08-26

**Authors:** Soraya van Etten, Lonneke Jansen, Michèlle Bal, Brian J. Dermody, Eggo Müller, John de Wit, Marijn Stok

**Affiliations:** 1 Department of Interdisciplinary Social Science, Faculty of Social & Behavioural Sciences, Utrecht University, Utrecht, The Netherlands; 2 Faculty of Geosciences, Copernicus Institute of Sustainable Development, Utrecht University, Utrecht, The Netherlands; 3 Department of Media and Culture Studies, Faculty of Humanities, Utrecht University, Utrecht, The Netherlands

**Keywords:** Food, Sustainability, Behaviour change, COM-B model, Adolescents, Socio-economic position, Intervention, Cohort study, Communities, Social networks

## Abstract

**Background:**

The present-day food system is a key driver of climate change and biodiversity loss, making it imperative for populations to shift towards more sustainable diets. The involvement of youth in this transition is vital because they are in a formative period where their identities, values, and norms, including their food behaviours, are being shaped. Special attention should be paid to youth in practical education because they are often overlooked in existing studies, yet evidence suggests they may lack the necessary resources to support dietary changes, resulting in lower levels of pro-environmental food-related behaviours. The aim of the FLY (Food-related Lifestyles in Youth) project is to study how sustainable food-related lifestyles and underlying factors develop in early adolescence, particularly in Dutch youth in practical education, how these spread in social networks, and to develop community-level intervention strategies to support youths’ transition to sustainable food-related behaviours.

**Methods/design:**

The FLY-project adopts a mixed-method approach. First, two literature reviews are conducted. A systematic review assesses how capabilities, opportunities and motivation are associated with sustainable food behaviours in youth, and how these elements interrelate in determining sustainable food-related lifestyles. A scoping review studies community-level interventions that target sustainable and healthy food-related behaviours. Second, focus groups are conducted to explore the barriers and facilitating factors concerning capabilities, opportunities, and motivations that Dutch youth in practical-level education experience to transition to more sustainable food-related lifestyles. Third, a cohort survey study is conducted to track the dynamic interplay between capabilities, opportunities, motivation, and changes in specific sustainable food behaviours over time, and to assess the diffusion of sustainable food-related lifestyles via social (media) networks. Fourth, an experimental research programme tests promising intervention approaches, some of which are co-created with youth, targeting relevant underlying factors.

**Discussion:**

This paper describes the rationale, conceptual framework, design and methods of the FLY-project. The FLY-project contributes to an understanding of underlying factors of sustainable food-related behaviours in adolescence and results in a multi-component intervention toolkit, with a particular focus on youth in practical education programmes.

**Supplementary Information:**

The online version contains supplementary material available at 10.1186/s40795-023-00757-2.

## Background

The current food system is a key driver of environmental degradation through pollution, deforestation, and loss of biodiversity, in addition to being a significant factor in the prevalence of health-related sustainability issues and non-communicable diseases [1]. Therefore, it is key that populations shift towards more sustainable diets by reducing or eliminating the consumption of animal-based products, increasing the consumption of plant-based, locally sourced and in-season foods, and minimizing food waste [1–3]. Furthermore, accepting and engaging with technological innovation (e.g., in-vitro meat) can play an important role in reducing the overall environmental impact of our food systems. By making these changes, individuals can not only benefit their health and well-being but also contribute to the global effort to mitigate climate change and preserve the planet for future generations [2, 4, 5].

Existing transition research strongly focuses on stakeholders (e.g., governments, food producers) and technological innovation in the food production system, leaving a dearth of behavioural science research into people’s willingness and ability to change their behaviour and the acceptance of innovation [2]. Yet, understanding why people engage in or resist more Sustainable Food-related Lifestyles (SFrL) is crucial for creating successful sustainable food transitions [3].

In research on sustainable food behaviour change, focusing on youth is important. Adolescents are in a critical period in life, when identities, norms, and values, including those related to food behaviours, are being formed [6] and when early adopters change their diets towards, for example, vegetarianism and veganism [7, 8]. Moreover, young people represent a large proportion of the total (food-related) consumption expenditure, have substantial influence over the market, influence directly or indirectly a large portion of family consumption, are more open to change, and their consumption patterns will have a lasting impact on their eating behaviour and that of their future children [9–13]. Despite this, young people are often ignored both in literature as well as in political decision-making related to sustainable behaviour [13].

In addition to *young* people not often being the focus of research, most studies to date have focused on people from higher socio-economic backgrounds [14, 15]. This is problematic because research suggests that individuals with a lower socio-economic position may not have access to the material and psychosocial resources typically available to those with a higher socio-economic position to support dietary changes [16]. Therefore, these groups are more likely to have more meat-based diets and generally show lower levels of pro-environmental food-related behaviours [17–19]. As a result, lower socio-economic groups have been left behind in the transition to SFrL practices, perpetuating disparities in health, environmental sustainability, and social justice.

In summary, there is a need for behavioural science research on SFrL to focus on youth from lower socio-economic backgrounds. The Food-related Lifestyles in Youth (FLY) project aims to fill this research gap by investigating both the transition to sustainable food behaviours in Dutch youth (12 to 16 years old) as well as the underlying factors influencing this process. By mapping adolescents’ social networks, the FLY project aims to address and mobilise youths’ social environment, building community-level motivation, capabilities and opportunities for engaging with the SFrL transition [20]. This paper outlines the conceptual framework underlying the FLY-project as well as the design and methods used.

### Definitions and conceptual framework

In line with common definitions of lifestyle [21], we define Sustainable Food-related Lifestyle (SFrL) as the whole of food-related behaviours that promote sustainability as well as the underlying mechanisms associated with those behaviours. Based on recent reports from the EAT-Lancet Commission [1] and the Intergovernmental Panel on Climate Change [3], we regard consuming locally sourced, and in-season foods, reducing or eliminating animal-based products, and minimizing food waste as the most critical sustainable food-related behaviours. We adopt the concept of lifestyle, which extends beyond behaviour, as it encompasses not only specific actions but also the underlying mechanisms influencing those behaviours, such as values, beliefs, and social norms, which are all interconnected [22]. Additionally, it recognizes that food choices are not solely driven by the need to nourish the body or personal taste preferences, but also serve as a means for individuals and groups to both express their identity and to affiliate or differentiate themselves from certain social groups [23]. In this study, SFrL is operationalized into measurable behaviours and related constructs by identifying and focusing on one or two of the most challenging and impactful sustainable food-related behaviours and the mechanisms underlying these specific behaviours.

In addition, this study operationalizes socioeconomic position based on the type of education that young people are enrolled in, with those in practical education programmes considered to have a lower socioeconomic position than those in theoretical education programmes. This operationalization was chosen for several reasons. First, education is a stable and robust indicator of socioeconomic position that is strongly linked to social class, status, and income [24]. Second, there is a strong association between education and (healthy) food-related behaviour, with research indicating that people with theoretical education levels have healthier diets than those with a practical level of education [25, 26]. Third, when studying adolescents, education is a more relevant indicator than income or occupational status because adolescents may not have fully entered the labour market yet. Finally, this operationalization offers practical advantages as the type of education is easy to measure [27] and it enables us to reach young people through schools, where they often form close-knit communities that influence identity and eating behaviours through social influence processes [28, 29].

Previous research on sustainable behaviour change has drawn on two main theories: Value-Belief-Norm (VBN) and the Theory of Planned Behaviour (TBP) [30, 31]. The VBN theory is a social psychology theory proposed by Stern et al. (1999) to explain people's pro-environmental behaviours. It emphasizes the role of values, sustainability-specific beliefs, and personal norms in shaping behaviour [32, 33]. The TPB is a widely recognized social psychology theory that proposes that behavioural intentions are influenced by people's attitudes, subjective norms, and perceived behavioural control [34–36]. Both theories foreground the role of individuals’ perceptions and motivational factors in guiding behaviour. However, concentrating only on motivation is insufficient for understanding why people engage in or resist sustainability transitions [37] and entails the risk of blaming those who struggle with the SFrL transitions for not keeping up [38]. The VBN theory and TPB both overlook the important role of structural factors, such as the social and physical environment, which are important in shaping dietary behaviour [39] and essential in driving systemic change towards sustainable food systems [2].

To gain a more comprehensive understanding of which factors shape SFrL in young people, particularly those in practical education, and how these develop over time, the FLY-project builds on the Capability-Opportunity-Motivation-Behaviour (COM-B) model [40]. The COM-B model proposes that behaviour is not only shaped by motivation but also by people's capabilities and opportunities. People's capabilities include their psychological and physical capacities to engage in a particular act, such as their knowledge, skills and self-efficacy. Opportunities encompass all factors in the physical or social environment that facilitate or hinder behaviour, such as the food environment and media environment. The COM-B model, which was originally developed in the realm of health behaviour change, offers a widely applicable integration of key perspectives on factors that shape behaviour, holding much promise to strengthen understanding of sustainable behaviour [41, 42]. Applying this model is particularly relevant when studying youth in practical education because the necessary capabilities and opportunities to transition to more SFrL are likely distributed unequally across socio-economic groups [38].

When studying SFrL and its underlying mechanisms, social networks are a key factor to consider. They are not just part of the Opportunities component of the COM-B model but an overarching concept that potentially affects all elements of the model. Social networks play a crucial role in shaping behaviour [43] and are fundamental for sharing knowledge, norms, values, and behaviours through social influence processes such as imitation, modelling, and conformity [44]. Furthermore, research has shown that social network characteristics are linked to dietary choices among young people [45]. Studies on the adoption of food technologies have also highlighted how characteristics of social networks are critical to the spread of information about innovation, the appreciation of its benefits and improvements, and the establishment of trust in the innovation's ability to meet expectations [46]. The FLY-project zooms in on the role of social networks in the SFrL transition by studying how capabilities, opportunities, motivation and behaviour spread through networks of young people.

Given their vital role in shaping behaviour, social networks are also considered to be the designated level for effective intervention delivery to achieve lasting changes in SFrL in youth [43, 47]. The COM-B model is particularly suitable for exploring effective intervention strategies at the community-level as it recognizes the interplay between individuals and their social and physical environments. Besides, the model is at the core of the innovative ‘behaviour change wheel’ approach, which is unique and effective in linking a wide range of factors that shape behaviour to multi-level intervention strategies [40]. By adopting a comprehensive approach to intervention strategies that encompasses more than individual behaviour change, we can capitalise on youth’s potential to drive change by engaging in a broad set of activities, including political participation and collective action [48].

### Aim and research questions

The overall aim of the FLY-project is to establish how a transition to more SFrL in youth, specifically in practical education, can be facilitated by a better understanding of how sustainable food behaviour and underlying factors develop in early adolescence and how these spread in social networks. The FLY-project adopts a mixed-method approach, combining literature reviews and focus groups with a quantitative cohort study and an experimental research programme in which interventions are piloted and evaluated, both quantitatively and qualitatively. Furthermore, part of the interventions is co-created with youth. Together, this ensures a rich and in-depth understanding of the barriers and facilitators of the SFrL transition in youth and enables the development of effective intervention strategies. We develop intervention strategies to support youths’ transition to sustainable diets by innovatively focusing on building community-level capabilities, opportunities, and motivation to adopt (more) sustainable behaviour. In collaboration with the Netherlands Nutrition Centre, we develop a multi-component intervention toolkit. As such, the research questions are threefold:

1. How do capabilities, opportunities, and motivation shape SFrL in young people, in particular those from lower socio-economic backgrounds?
2. How do capabilities, opportunities, motivation and behaviour evolve over time in social networks of young people?
3. What are effective community-level intervention strategies to facilitate the SFrL transition in youth?

## Methods

### Study design

**Fig. 1:**
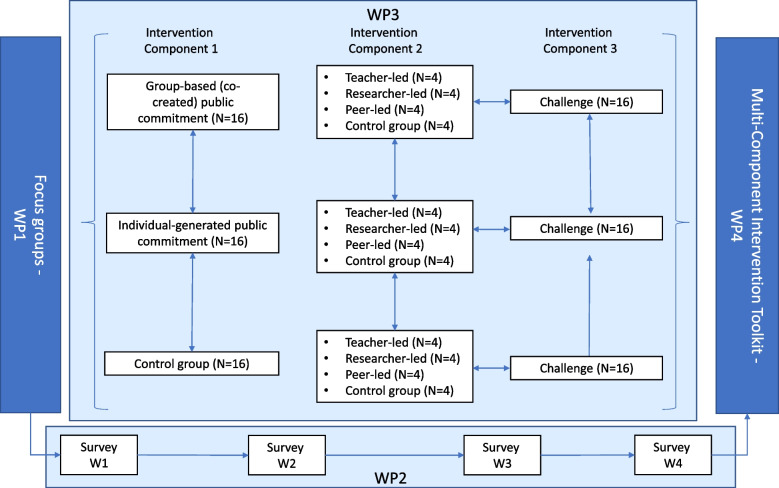
Overview of the research design

Figure 1 presents an overview of the research design. A SPIRIT checklist is presented in Additional file 1. First, two literature reviews are conducted to explore the role of capabilities, opportunities and motivation of youth towards sustainable food-related lifestyles, and to ensure state-of-the-art intervention strategies are applied in later stages of the project, respectively. Second, focus groups are conducted to provide rich qualitative insights into the capabilities, opportunities, and motivations important for the SFrL transition. Third, we conduct a cohort study to track the dynamic interplay between capabilities, opportunities, motivation, and changes in specific sustainable food behaviours over time, and to assess the diffusion of SFrL via social (media) networks. Fourth, an experimental research programme tests intervention approaches, targeting relevant COM-B elements. Lastly, a synthesis of the findings from the reviews, focus groups, cohort study and experimental research programme feed into the development and implementation of a multi-component intervention toolkit.

**Table 1:** Schedule of enrolment, interventions, and assessments

	**Study Period**									
	**Enrolment**	**Allocation**	**Post-allocation**							**Close-out**
**Timepoint**	***-t***_***1***_	**0**	***t***_***1***_	***t***_***2***_	***t***_***3***_	***t***_***4***_	***t***_***5***_	***t***_***6***_	***t***_***7***_	***t***_***8***_
**ENROLMENT:**										
**Eligibility screen**	X									
**Informed consent**	X									
**Allocation**		X								
**INTERVENTIONS:**										
***Public commitment***				X						
***Intervention leader***						X				
***Co-creation***								X		
**ASSESSMENTS:**										
***Sustainable food-related lifestyles and related COM-B elements***			X		X		X		X	X

### Participants and recruitment

Recruitment of participants is undertaken in collaboration with the municipality of Utrecht and the Netherlands Nutrition Center (NNC). The target group consists of young people between the ages of 12 and 16 who reside in or attend school in the Utrecht region. The specific recruitment methods vary depending on the study being conducted and are described below.

### Literature reviews

The first systematic literature review synthesizes the available evidence regarding the associations of capabilities, opportunities and motivation with SFrL in young people, and the interrelations between these COM-B elements in determining sustainable food-related lifestyles. The databases MEDLINE (Ovid), EMBASE (Ovid), PsycINFO (Ovid) and Web of Science are searched for relevant publications. The search terms include synonyms of sustainable food-related lifestyle, the COM-B model and young people (people under the age of 26). These synonyms are combined with the main Boolean operators “OR” and “AND” into one comprehensive search strategy. Studies are selected by screening titles and abstracts first, followed by full-text screening by applying selection criteria. Studies included are experimental (e.g., laboratory, field, randomized controlled trials) and observational studies (e.g., focus groups, questionnaires, case studies). Studies with adult populations or non-consumers (e.g., farmers, restaurant owners) are excluded, as well as reviews, meta-analyses, background articles and unpublished studies (e.g., conference abstracts, trial protocols). Reference lists of relevant reviews and included studies are hand-screened for potential studies that meet the inclusion criteria. The titles and abstracts of 10% of the total number of studies are screened by a second author independently. Any discrepancies between authors are adjudicated by a third author. From the articles that are included, the following data is abstracted: author, publication year, year(s) data collected, country, type of article/study design, theoretical approach, sample characteristics (e.g., sample size, mean age, percentage of women), description of factors measured in the study, target behaviour(s), measured outcome, results/conclusions.

A scoping literature review synthesizes evidence regarding community-level interventions that target healthy or sustainable food-related behaviours. The aim is to ensure that state-of-the-art intervention strategies are applied in the following stages of the project. The search terms include synonyms of community-level intervention, youth, and healthy or sustainable food-related behaviour. These synonyms are again combined with the main Boolean operators “OR” and “AND” into one comprehensive search strategy. The same databases as in the first literature review are searched and screening and selection of articles and abstraction of data follow the same systematic approach.

### Focus group discussions

Twelve focus group discussions (FGDs) with six to eight participants are planned. The actual number of FGDs will depend on reaching data saturation, which depends on various parameters of saturation, including group composition, code characteristics, and desired type and degree of saturation [49]. High school students between the ages of 12 and 16 enrolled in various educational programmes, including practical and theoretical programmes, are recruited through football clubs in the Utrecht region. Boys, girls and mixed-gender teams are recruited. This approach aims to ensure a more targeted and comfortable discussion environment for participants. Participants vary in ethnicity and socio-economic background within the groups to ensure variation in capabilities, opportunities and motivation for different sustainable food-related behaviours. FGDs take place at a time and location convenient to participants to minimise burden and take no more than 1.5 h each. The moderator uses prompts to start the discussion on sustainable food in general, as well as perceived capabilities, opportunities, and motivations for sustainable food-related behaviours specifically. A semi-structured discussion guide is developed to ensure consistency in questions asked across groups. Discussion guides are pretested in a separate sample of youth, including cognitive interview testing to ensure concepts are understood. All FGDs are recorded and transcribed verbatim, and thematic analysis is conducted using NVivo. Coding is derived from the data, by first familiarization of the data by revisiting the focus group discussion audio and verbatim transcript, followed by code generation. Inter-coder agreement is assessed between two researchers to compare the consistency of code use and rectify discrepancies before the whole data set is coded. A set of overarching themes are developed, including theme definition and naming, after which discussion takes place to refine the way the themes are developed. The FGDs are used to explore the barriers and facilitating factors concerning capabilities, opportunities, and motivations that youth experience to transition to more SFrL. The results obtained from the FGDs are utilized to select one or two specific sustainable food-related behaviours (e.g., animal protein intake reduction, avoidance of food waste). These selected behaviours reflect the ones that young people encounter the most barriers in adopting, the behaviours deemed most important by youth, those that are feasible to change, and those that have a significant impact on the climate crisis. Subsequently, these behaviours are subjected to further examination in the cohort study and tested through pilot interventions.

### Cohort study

The sustainable food-related behaviours selected during the focus groups are investigated in more depth in the cohort study consisting of four consecutive waves of data collection undertaken over two years (i.e., bi-annually). Based on existing research (synthesised in the first systematic literature review) and insights from the focus groups, a survey is developed in which self-reported capabilities, opportunities, motivations, and food-related behaviours are measured along with socio-demographic variables (e.g., age, gender; only in survey wave 1). Surveys are conducted online using Qualtrics and the estimated time to conduct each survey is 30 min. The survey is pretested in a separate sample of youth aged between 12–16, to ensure concepts and questions are understood. The survey can be filled out in class or during another time convenient for the participants, to minimise the burden on participants and teachers. Recruitment of cohort members takes place through practical-education-level secondary schools in the Utrecht region, specifically from first- and second-year classes (to ensure that cohort members can participate in all waves of data collection during secondary education). Interrelations between capabilities, opportunities, motivations, and sustainable food-related behaviours are analysed cross-sectionally in wave 1 and longitudinally from waves 2–4 via Structural Equation Modeling (SEM). As participants are nested in friend groups, which are nested in school classes, which in turn are nested in schools, multilevel analyses are conducted. Also, the social networks (including in-school, out-of-school, and social media environments) of respondents are mapped using questionnaires to map ego networks, showing how respondents are embedded within networks and identifying central group members within school classes. The development of social networks over time is also investigated. The specific analyses mentioned however may change based on the nature of the data and other factors.

### Intervention component pilot testing 

Table 1 presents an overview of the schedule of enrolment, intervention component pilot testing and assessments. Intervention components are developed and tested to target sustainable food-related behaviours and the barriers and facilitators related to these behaviours, which can be rooted in either capabilities, opportunities, or motivation (or any combination of these three). In a stepwise research programme we explore the effects of three intervention components. The specific targeted behaviours, underlying mechanisms, and intervention components will be determined based on the findings from the literature review, focus groups, and survey conducted as part of the research programme. However, to explain the design, we will provide three plausible examples of intervention components, based on an initial screening of the literature: public commitments, peer-led interventions, and co-created challenges.

First, we may investigate the effect of public commitments. There are three conditions: publicly voicing a personal (individually created) commitment versus a group-based (co-created) commitment versus a no-commitment control condition on sustainable food-related behaviour and underlying capabilities, opportunities, and motivations. The content of the commitments varies according to the specific factors associated with sustainable food-related behaviours. A first study tests the effects of the different commitment conditions on capabilities, opportunities, motivations, and sustainable food-related behaviours in a lab-based experiment. A second study tests these effects in a field experiment amongst young people who do not participate in the cohort study. Power calculations estimating medium-size effects, using power = 0.80 and α = 0.05 indicate that a minimum of *N* = 160 participants should be included in each of these studies. Participants for these pilots are recruited through targeted advertising through relevant channels (e.g., sports clubs and social media). In a third study, this intervention component is delivered to the 48 classes participating in the cohort study. We assess the effects of this intervention component through analysis of the second survey wave.

Second, we may investigate the effect of who leads a targeted intervention. The intervention will focus on identified barriers and facilitators, for instance strengthening cooking skills through a cooking class, or making personal motivation salient in a class discussion on personally relevant reasons for making sustainable food-related choices. There are four conditions: teacher-led versus expert-led versus peer-led versus no-intervention control. In both the teacher-led and the expert-led conditions, the interventions are designed by the research team in collaboration with the NNC. In the peer-led condition, ambassadors of the different classes come together to co-create an intervention with the research team and the NNC. Ambassadors are young people who play a central role in classes, as identified through social network analysis of the first two waves of survey data. Again, the pilot testing consists of two experiments amongst youth from the target population (using vignettes wherein the messenger identity is varied) and a third study in the cohort (with effects assessed through analysis of the third survey wave). For the two pilot studies, power calculations indicate that a minimum of *N* = 180 participants should be included in both pilots.

Third, we may investigate the effect of co-creation on the effectiveness of an intervention. We make use of a challenge-based approach, asking all 48 classes to develop an effective intervention to promote the adoption of SFrL. This intervention component again focuses on identified barriers and facilitators, and could, for instance, strengthen psychological capabilities through a challenge related to the question “How can we increase knowledge?”, or increase physical opportunities by focusing on the question “How can we create a more sustainable food environment?”. We undertake a qualitative process evaluation by monitoring the intervention development process in a subset of the classes and evaluating the designed interventions against the criteria of systematic intervention development. We use the fourth wave of the survey to test the effects of co-creation on SFrL and network evolvement. To encourage engagement with the challenge, we reward the three best interventions with a monetary prize (€2.000, €1.000, and €500, respectively) to enable the implementation of the proposed intervention in the respective schools under the supervision of the research team.

### Developing and pilot testing a multi-component intervention toolkit

Finally, the findings from the literature reviews, focus groups, cohort study and pilot tests are synthesized to develop a multi-component intervention toolkit promoting SFrL in youth with practical-level education. This toolkit is developed in collaboration with NNC and combines the community-level intervention components that were found most effective in the pilot tests. Guided by a logic model of change, the toolkit addresses specific capabilities, opportunities, and motivations related to the targeted sustainable food-related behaviours. It evolves as the project progresses and is finalised based on the synthesis of all findings. In addition, a multi-component intervention is developed and pilot-tested in the four control group classes.

## Discussion

The FLY-project is a mixed-method research project that combines literature reviews, focus groups, a quantitative cohort study and an experimental research programme, including the co-creation of interventions with youth, that aims to provide a rich and in-depth understanding of the barriers and facilitators of the SFrL transition in youth and facilitate the development of effective community-level interventions strategies. As such, the project is a valuable contribution towards achieving the SFrL transition in the Dutch population and to the emerging social science scholarship into sustainability transitions in general and SFrL in particular.

### Limitations and strengths of the project

A key strength of the FLY-project is its innovative mixed-methods and co-creation approach. Combining qualitative, quantitative and experimental research, as well as social network analysis, allows us to simultaneously undertake fundamental and applied research and guarantees that findings have both high internal and external validity, and are fit for practice. The co-creation approach ensures that the interventions are tailored to the specific needs and preferences of young people, which is expected to increase the effectiveness of the interventions. Besides, it allows youth to actively participate in the research process, providing them with a valuable learning experience, and the opportunity to contribute their perspectives and to develop a wide range of skills [20].

Second, the project innovatively applies the COM-B model, broadening research into sustainable behaviour change beyond the currently dominant motivational accounts. This model allows us to take into account the broader social and physical environment in which youth are situated, including the power dynamics and structural inequalities that shape food systems. It also enables us to develop intervention strategies that go beyond individual behaviour change and recognize the potential of young people to become agents of change in their community, creating a ripple effect. This is important because systemic change can only occur when other stakeholders, such as schools, (local) governments and companies, take action as well [2].

One of the main challenges to be anticipated in the FLY-project is recruiting sufficient participants to meet the sample size and power requirements of the studies, taking into account potential loss to follow-up over the course of the (cohort) study. Participant loss to follow-up may be especially prevalent when studying young people, due to for example time constraints or logistical issues. This would have negative implications for the validity and reliability of the study. To increase recruitment and minimize the drop-out of participants, we implement several strategies, such as providing incentives, establishing good communication with participants, and making participation as convenient and accessible as possible. Furthermore, the intended collaboration with schools and other organizations, such as the NNC and the municipality of Utrecht, is expected to enhance participation and retention.

Second, it is important to acknowledge that studying disadvantaged groups, such as youth from lower socio-economic positions, involves the risk of reproducing oppressive power dynamics [50]. One particular risk is undervaluing or dismissing the knowledge and skills possessed by young people and their communities, which could perpetuate negative biases and stereotypes [50]. To prevent this, a co-creation research approach is adopted, where young people are involved in the research process and the development of interventions. In addition, it is important for the researchers to continuously reflect on their own identities, experiences, and biases, and consider how they may impact the research, and how to mitigate these effects [51]. The study also adopts an intersectional approach, acknowledging that the experiences of young people from lower socio-economic positions are shaped by a range of social, cultural, and economic factors, rather than treating them as a homogenous group [52].

A further potential limitation of the study is that the findings may have limited generalizability to other contexts outside the Netherlands. When interpreting and applying the study's results, it is crucial to consider the potential impact of location-specific factors. Furthermore, future research should explore how the study's findings can be adapted and applied in diverse food systems and cultures to maximize their relevance and applicability.

## Conclusion

The FLY-project's emphasis on community-level intervention components, co-created with youth, as well as its innovative use of the COM-B model and mixed-methods approach, ensures valuable contributions to SFrL transition research and practice. Several strategies are employed to address potential challenges, such as participant loss to follow-up. The project has the potential to empower young people to become agents of change in their communities and contribute to systemic change in the food system. By producing a multi-component toolkit for community-level intervention, the project can potentially influence young people with practical-educational backgrounds at a national level. This impact is likely to last throughout their lives, as adolescence has proven to be an important formative life phase for developing healthy and sustainable diets.

### Supplementary Information

**Additional file 1. **

## Data Availability

Data storage will comply with the Data Storage (Archiving) Protocol (2016) of the Faculty of Social and Behavioural Sciences, Utrecht University. Data collected through the longitudinal panel study and experimental studies will be made available (under restricted access) to other bona fide researchers through YoDa. The audio recordings from the focus groups will not be made available to the public, since these will not be retained after transcripts are made. Any other data that contains personal data will also not be made publicly available.

## Abbreviations

FLY: Food-related lifestyles in Youth
SFrL: Sustainable Food-related Lifestyles
VBN: Value-Belief-Norm
TBP: Theory of Planned Behaviour
COM-B: Capability-Opportunity-Motivation-Behaviour
NNC: Netherlands Nutrition Center
FGDs: Focus Group Discussions
SEM: Structural Equation Modeling
